# Associations between mental health and disease severity in individuals with psoriasis

**DOI:** 10.1093/skinhd/vzaf108

**Published:** 2026-01-28

**Authors:** Eva Fleischmann, Melanie Lenger, Frederike T Fellendorf, Nina Dalkner, Eva Z Reininghaus, Katja Großschaedl, Wolfgang Weger, Thomas Graier, Wolfgang Salmhofer, Rainer Hofmann-Wellenhof, Peter Wolf

**Affiliations:** Department of Psychiatry and Psychotherapeutic Medicine, Medical University of Graz, Graz, Austria; Department of Psychiatry and Psychotherapeutic Medicine, Medical University of Graz, Graz, Austria; Department of Psychiatry and Psychotherapeutic Medicine, Medical University of Graz, Graz, Austria; Department of Psychiatry and Psychotherapeutic Medicine, Medical University of Graz, Graz, Austria; Department of Psychiatry and Psychotherapeutic Medicine, Medical University of Graz, Graz, Austria; Department of Dermatology and Venereology, Medical University of Graz, Graz, Austria; Department of Dermatology and Venereology, Medical University of Graz, Graz, Austria; Department of Dermatology and Venereology, Medical University of Graz, Graz, Austria; Department of Dermatology and Venereology, Medical University of Graz, Graz, Austria; Department of Dermatology and Venereology, Medical University of Graz, Graz, Austria; Department of Dermatology and Venereology, Medical University of Graz, Graz, Austria

## Abstract

**Background:**

Psoriasis and mental health are closely intertwined; however, certain aspects remain underexplored. Thus, continued research on this topic is important to deepen our understanding.

**Objectives:**

To explore the mental health of individuals with psoriasis – specifically depressive symptoms, mental functioning, stress symptoms and coping strategies – and to find associations with physical manifestations of psoriasis.

**Methods:**

A cross-sectional sample of 214 individuals with psoriasis (107 with illness duration >16 years and 107 with illness duration ≤16 years; cut-off: median) completed the Beck Depression Inventory-II, the SF-12 measuring mental and physical functioning, and the Stress and Coping Inventory. Additionally, the Psoriasis Area and Severity Index (PASI) was assessed. The study was conducted during the COVID-19 pandemic.

**Results:**

Individuals with psoriasis reported worse mental and physical health than the general population (norm sample of SF-12) but did not differ significantly from each other. While individuals with illness duration >16 years had lower psoriasis severity than those with illness duration ≤16 years, mean PASI scores indicated low illness severity overall. PASI scores did not show significant correlations with mental health inventories in either group.

**Conclusions:**

Individuals with psoriasis reported lower mental health during the COVID-19 pandemic than a healthy, prepandemic norm sample, independent of psoriasis severity. Age and illness duration seem to facilitate coping with the disease. Nevertheless, individuals with psoriasis are in need of continued mental health support, regardless of illness duration.

## Teaser

This study’s aim was to research psoriasis patients' mental state and its relation to disease severity. It is shown that while individuals affected by psoriasis seemed to have worse mental health than the norm sample during the COVID-19 pandemic, there was no significant association between mental health and psoriasis severity. Nevertheless, mental health support for affected individuals is highly recommended, especially for young individuals.

What is already known about this topic?

- At present, we lack in-depth knowledge about the mental state of patients with psoriasis and its relation to disease severity.

What does this study add?

- This study shows that while individuals affected by psoriasis seemed to have worse mental health during the COVID-19 pandemic than the norm sample, the relationship between disease severity and mental state is complex and might be strongly influenced by external factors.
- Mental health support for affected individuals is highly recommended, especially for younger patients.

Psoriasis is an inflammatory dermatological disease affecting 1.5–2% of the Western population.^1^ Its aetiopathogenesis is multifactorial, including genetic,^2^ immune-mediated^3,4^ and psychosocial factors.^5^ Clinical presentation typically involves erythematous plaques covered with silver scales, affecting most often the knees, elbows, the lumbosacral region and the scalp.^6^ The course of the disease is often chronic,^7^ and common comorbidities include arthritis, cardiometabolic diseases and inflammatory bowel diseases.^6^ Moreover, patients with psoriasis face a considerable risk of psychiatric comorbidities, including vascular dementia, schizophrenia, affective disorders and generalized anxiety disorder.^8^ In sum, psoriasis imposes a high burden on affected individuals and the healthcare system due to treatment costs, reduced psychosocial functioning and decreased work ability.^9,10^

As the skin is the largest organ of the human body and directly observable to others, psoriasis plaques are often visible and recognizable. In many patients, this results in stigmatization^11,12^ and significant psychological burden.^13,14^ Additionally, severity, pruritus and pain play significant roles in physical and mental wellbeing.^15,16^ Psoriasis is related to feelings of shame^17^ and anxiety,^18^ self-consciousness,^19^ depressive mood,^20^ impaired sexuality,^21^ reduced wellbeing and diminished quality of life.^16^ Affective symptoms may manifest as emotional instability and negative emotional reactions.^5,22^

Research has demonstrated that disease severity, most commonly measured with the Psoriasis Area and Severity Index (PASI^23^), negatively impacts physical and mental wellbeing and^15,16^ quality of life,^15,24^ as well as psychosomatic conditions.^15^ Wintermann *et al*. found that more pronounced symptoms of depression and were associated with reduced improvement in psoriasis severity during dermatological treatment.^25^ Moreover, studies have shown that patients with psoriasis exhibit alexithymia^15,26^ and attachment-related avoidance more frequently than inpatients with other dermatological conditions.^27^ Alexithymia, in particular, appears to be a risk factor, as individuals with psoriasis and alexithymia report lower quality of life,^28,29^ as well as more symptoms of anxiety and depression.^28^

These mental conditions can contribute to body dysmorphic concerns^30^ and even to body dysmorphic disorder, significantly impairing daily functioning.^31^ Avoidant behaviour, social withdrawal, obsessively covering affected body areas and experiencing stress are risk factors for developing mental disorders.^32^ Conversely, stress caused by psychosocial or life events, psychological conditions or somatic disorders also exacerbates psoriasis symptoms.^33–35^ A study researching mice found that the upregulation of the cytokine interleukin (IL)-1β and the IL-23p40 subunit of IL-12 and IL-23 substantially contributes to psoriasis aggravation under stress.^36^

Given the limited number of studies researching psoriasis severity with regard to mental health, the aims of the current study were to (i) explore the psychological profile of individuals with psoriasis (including mental health, depressive symptoms and stress, with the variables overall burden, stress symptoms, positive thinking, active coping, social support, religious faith and alcohol/smoking) and to (ii) find associations between current psoriasis severity and mental state. As the results might depend on illness duration and could impact treatment, group effects were analysed for illness duration ≤16 years and >16 years. It was hypothesized that current psoriasis severity would correlate negatively with mental health and positively with depressive symptoms and stress.

## Patients and methods

### Participants and procedures

Patients (aged 18 years and older) with chronic plaque-type psoriasis presenting at the Psoriasis Outpatient Clinic of the Department of Dermatology and Venereology, Medical University of Graz, were enrolled in the study after providing informed consent. The cross-sectional assessment was conducted between September and December 2021 and included sociodemographic parameters, illness and medication history, psoriasis severity according to the PASI,^23^ and current psychosomatic symptomatology measured with validated self-report scales. With written consent, participants completed the self-report scales on a tablet prior to dermatological examination. In sum, 254 individuals with psoriasis were recruited, 214 of whom had complete datasets and were included in the final sample. A priori sample size calculation was not performed.

### Measurements to assess the psychological profile of individuals with psoriasis

Several standardized questionnaires, administered in German, were used to measure psychological parameters and psoriasis severity, and their associations with mental health.

The PASI assesses the severity of psoriasis by evaluating both the percentage of body surface area affected and symptom severity (redness, plaque thickness and scaling). Scores range from 0 to 72, with higher values indicating greater severity. A score of 0–10 indicates mild psoriasis, while a score of ≥11 indicates moderate-to-severe psoriasis.^23^

The SF-12^37^, derived from the SF-36,^38^ was used to measure health status and its impact on self-rated quality of life in the past 4 weeks. Consisting of 12 questions belonging to 8 health dimensions, it yields 2 summary scores: (i) the Physical Component Summary (PCS-12), assessing general health, physical functioning, role limitations due to physical health impairments and bodily pain; and (ii) the Mental Component Summary (MCS-12), measuring mental health (psychological wellbeing and distress), social functioning, role limitations due to emotional problems and vitality (energy/fatigue). Both scores range from 30 to 70, with higher values indicating higher health-related quality of life. For example, the domain General Health is assessed by presenting the following question: ‘In general, would you say that your health is poor/fair/good/very good?’^39^ Cronbach's alpha values of 0.72–0.89 for both subscales are considered acceptable.^40^ The results of the current study were compared with the SF-12 manual's norm sample of individuals aged 45–54 years, using *t*-tests.

The Beck Depression Inventory-II (BDI-II) is a 21-item ­self-report questionnaire quantifying depressive symptomatology during last 2 weeks. Total scores range from 0 to 63. A score of up to 8 indicates no depression, 9–13 minimal depression, 14–19 mild depression, 20–28 moderate depression and 29–63 severe depression. Internal consistency (Cronbach's α ≥ 0.84) and reliability (*r* ≥ 0.75) are satisfactory.^41^

The Stress and Coping Inventory-II (SCI) is a 54-item questionnaire with 9 subscales to measure stress levels, stress symptoms and coping strategies during the past 3 months. The three stress-related subscales include stress through insecurity, stress through overload and perceived threats related to different areas of life (e.g. work, health and living situation). The seven items of each subscale are answered by means of a 7-point Likert-like scale (1 = no strain, 7 = high strain), yielding scores between 7 and 49. These three subscales are combined to calculate the overall burden score, ranging from 21 to 147, with higher scores reflecting greater overall stress burden.

The stress symptoms subscale includes 13 items assessing stress symptoms of somatic (e.g. sleep problems) and psychological nature (e.g. difficulties in concentration; range 13–52). Coping includes five subscales, each consisting of four items and ranging from 4 to 16 points: positive attitude, active management strategies, social support, alcohol and cigarette consumption, and support through religious faith. The latter six subscales are rated on a 4-point Likert-like scale (1 = does not apply, 4 = applies). The SCI has Cronbach's alpha values from 0.69 to 0.88.^42^

### Statistical methods

IBM SPSS software (https://www.ibm.com/de-de/spss) was used for all statistical analyses. The sample was divided into two groups via median split (cutoff 16 years), using the variable illness duration of psoriasis. Normality was tested with Kolmogorov–Smirnov tests. Except for age, all metric variables were not normally distributed; however, according to the central limit theorem, distributions approximate normality with sample sizes ≥30.^43^ Outliers were considered to be naturally occurring and were included in the sample, as they did not to substantially distort the results. To test for group differences between individuals with illness duration ≤16 years and >16 years, a *t-*test for age, χ^2^ tests for sex, somatic and psychiatric comorbidity, intake of psoriasis medication and antidepressants, as well as smoking, and a Fisher's exact test for educational level were calculated. The MCS-12 and PCS-12 values of both groups were compared with the norm sample of individuals aged 45–54 years, using *t-*tests.

A Mancova was conducted to compare parameters of illness duration, physical health (PASI, PCS-12) and mental health (MCS-12, BDI-II and SCI subscales), with age, sex and education entered as covariates. Levene's tests were nonsignificant for all variables except PASI, psoriasis duration and the SCI subscale alcohol/smoking.

Partial correlation analyses controlling for age and education were used to investigate the relationship between the PASI and the following variables in individuals with both illness duration groups (≤16 years and >16 years): BDI-II, the SCI subscales (MCS-12 and PCS-12) and the SCI subscales (overall burden, stress symptoms, positive thinking, active coping, social support, religious faith and alcohol/smoking). Education was used as a covariate when calculating correlations of age with PASI, MCS-12 and PCS-12. As sex did not relate significantly to any variables, it was not included as a covariate. The false discovery rate (FDR) procedure was used to control for Type I error.

## Results

### Sample description

The sample consisted of 214 individuals diagnosed with psoriasis, divided by median split (cutoff 16 years) according to psoriasis duration (see Table 1). Most participants were taking biologics at the time of the survey and did not have any diagnosed psychiatric comorbidities. The mean PASI score was low in most participants, aside from a few outliers. Individuals with illness duration >16 years were, on average, older, more frequently treated with biologics, had lower PASI scores, reported more somatic comorbidities and smoked less often than individuals with illness duration ≤16 years. The groups did not differ in psychological parameters, as indicated by a Mancova including SF-12, BDI-II and PASI scores, and illness duration as dependent variables, with age, sex and education as covariates [*F*(14, 196) = 27.73, *P* < 0.001, η^2^ = 0.67].

**Table 1:** Sociodemographic and psychological characteristics of 214 individuals with psoriasis, comparing individuals with a psoriasis duration ≤16 years and >16 years

Variable	Psoriasis duration ≤ 16 years (*n* = 107)	Psoriasis duration > 16 years (*n* = 107)	Statistics	*P*-value	Effect size
Age (years), mean (SD)	43.00 (15.00)	53.00 (13.00)	***t*(208.33) = −5.36**	**<0**.**001**	0.73
Sex					
Male	60 (56.1)	65 (60.7)	χ^2^(1) = 0.48	0.49	0.05
Female	47 (43.9)	42 (39.3)			
Education level			5.133a	0.27	0.16
None	4 (3.7)	0 (0.0)			
Secondary school	56 (52.3)	63 (58.9)			
High school/college	34 (31.8)	28 (26.2)			
Bachelor's degree	5 (4.7)	7 (6.5)			
Master's degree	8 (7.5)	9 (8.4)			
Somatic comorbidity			**χ^2^(1) = 6.69**	**0.01**	0.18
Yes	28 (26.2)	46 (43.0)			
No	79 (73.8)	61 (57.0)			
Psychiatric comorbiditiy			χ^2^(1) = 0.48	0.49	0.05
Yes	9 (8.4)	12 (11.2)			
No	98 (91.6)	95 (88.8)			
Intake of antidepressants			χ^2^(1) = 0.27	0.60	0.04
Yes	7 (6.5)	9 (8.4)			
No	100 (93.5)	98 (91.6)			
Smoking			**χ^2^(1) = 9.49**	**0.002**	0.21
Yes	53 (49.5)	31 (29.0)			
No	54 (50.5)	76 (71.0)			
Illness duration (years), mean (SD)	7.75 (4.95)	31.44 (10.41)	***F*(1, 209) = 324.59**	**<0.001**	0.63
PASI, mean (SD)	2.30 (4.60)	1.20 (2.50)	***F*(1, 209) = 6.80**	**0.01**	0.03
PCS-12, mean (SD)	41.52 (5.18)	41.25 (4.95)	*F*(1, 209) = 0.98	0.32	0.01
MCS-12, mean (SD)	47.25 (6.67)	47.59 (5.91)	*F*(1, 209) = 0.60	0.45	0.00
BDI-II, mean (SD	5.24 (6.88)	5.62 (8.88)	*F*(1, 209) = 1.11	0.29	0.01
SCI overall burden, mean (SD)	55.65 (13.09)	53.27 (14.93)	*F*(1, 209) = 0.03	0.88	0.00
Stress symptoms	32.54 (5.68)	32.50 (6.11)	*F*(1, 209) = 1.64	0.20	0.01
Positive thinking	14.21 (1.77)	14.31 (1.88)	*F*(1, 209) = 0.00	0.95	0.00
Active coping	14.50 (1.73)	14.52 (1.94)	*F*(1, 209) = 0.03	0.86	0.00
Social support	15.27(1.68)	15.26 (1.47)	*F*(1, 209) = 0.10	0.76	0.00
Faith	11.03 (2.26)	11.50 (2.32)	*F*(1, 209) = 0.16	0.69	0.00
Alcohol/smoking	10.94 (1.59)	10.54 (1.42)	*F*(1, 209) = 2.39	0.12	0.01

Results are presented as *n* (%) unless otherwise specified. Significant results are in bold. BDI-II, Beck Depression Inventory-II; MCS-12, Mental Component Summary of the SF-12; PASI, Psoriasis Area and Severity Index; PCS-12, Physical Component Summary of the SF-12; SCI, Stress Coping Inventory. ^a^Fisher's exact test was used.

**Figure 1:**
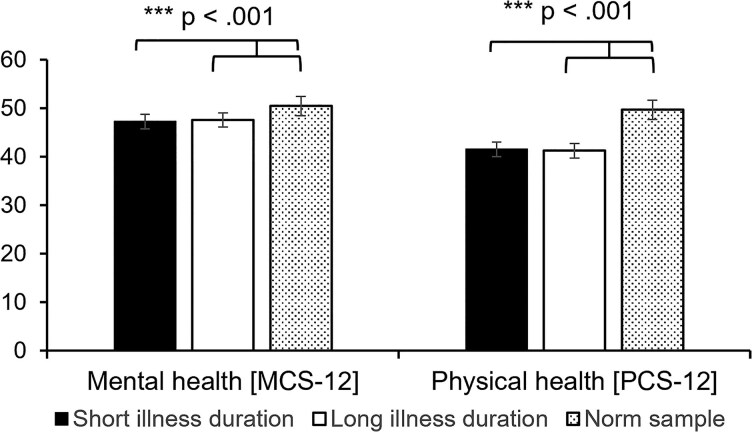
Mental and physical health in individuals with a psoriasis duration of >16 years and ≤16 years, as well as the norm sample. MCS-12, Mental Component Summary of the SF-12; PCS-12, Physical Component Summary of the SF-12. ***P < 0.001.

In comparison with the norm sample of individuals aged 45–54 years, our sample had significantly lower mean (SD) PCS-12 scores [see Figure 1; norm sample: 49.71 (9.50), *n* = 324; illness duration ≤16 years: *t*(336.157) = 11.26, *P* < 0.001; illness duration >16 years: *t*(350.516) = 11.88, *P* < 0.001] and MCS-12 scores [norm sample: 50.45 (9.55), *n* = 324; illness duration ≤16 years: *t*(259.132) = 3.83, *P* < 0.001; illness duration >16 years: *t*(295.519) = 3.67, *P* < 0.001].^39^ The groups did not differ in medication intake except for the number of biologics: individuals with illness duration >16 years were taking significantly more biologics compared with those with illness duration ≤16 years (see Table 2).

**Table 2:** Medication used to treat psoriasis in 214 individuals with a psoriasis duration ≤16 years and >16 years

Medication	Psoriasis duration ≤ 16 years (*n* = 107)	Psoriasis duration > 16 years (*n* = 107)	χ^2^(1)	*P*-value	Effect size
Topical treatments					
Calcineurin inhibitors	1 (0.9)	4 (3.7)	1.84	0.18	0.09
Combination product (corticosteroid, vitamin D analogue)	23 (21.5)	26 (24.3)	0.24	0.63	0.03
Corticosteroids	7 (6.5)	8 (7.5)	0.07	0.79	0.02
Vitamin D analogues	33 (30.8)	27 (25.2)	0.83	0.36	0.06
Other	4 (3.7)	2 (1.7)	0.69	0.41	0.06
Systemic treatments					
Biologics^a^	77 (72.0)	89 (83.2)	**3.87**	**<0.05**	**0.13**
Dimethylfumarate	0 (0)	2 (1.7)	2.02	0.16	0.10
JAK inhibitors	3 (2.8)	2 (1.7)	0.20	0.65	0.03
Methotrexate	0 (0)	3 (2.8)	3.04	0.08	0.12
Phosphodiesterase-4 inhibitors	6 (5.6)	2 (1.7)	2.08	0.15	0.10

Data are presented as n (%) unless otherwise stated. Significant P-values are in bold. JAK, Janus kinase. ^a^Biologics included tumour necrosis factor-α inhibitors (adalimumab, etanercept, golimumab), interleukin (IL)-12/23 inhibitors, IL-17 inhibitors (brodalumab, ixekizumab, secukinumab) and IL-23 inhibitors (guselkumab, risankizumab).

### Correlations between psoriasis severity and psychological wellbeing

Partial correlations controlling for education showed that PASI was not significantly associated with age in either illness duration group. Controlling for both education and age, PASI showed a significant negative correlation with MCS-12 in individuals with illness duration >16 years. Beyond this, no significant correlations emerged between PASI and illness duration, PCS-12 or the variables assessing mental health (MCS-12 in individuals with illness duration ≤16 years, BDI-II and the SCI subscales: overall burden, stress symptoms, positive thinking, active coping, social support, religious faith and alcohol/smoking) after FDR use (see Table 3). Correlations between age and PCS-12 were significant in individuals with illness duration ≤16 years (*r* = −0.31, *P* = 0.001) but not illness duration >16 years (*r* = −0.22, *P* = 0.03) after FDR correction. Correlations between age and MCS-12 did not remain significant after FDR correction in either group (illness duration ≤16 years: *r* = 0.24, *P* = 0.01; illness duration >16 years: *r* = 0.20, *P* = 0.04).

**Table 3:** Correlations between the Psoriasis Area and Severity Index (PASI) and variables assessing mental health status in 214 individuals with psoriasis duration ≤16 years and >16 years

Variable	Psoriasis duration ≤16 years (*n* = 107)		Psoriasis duration >16 years (*n* = 107)	
	*r*	*P*-value	*r*	*P*-value
Age	0.14	0.16	0.11	0.25
Illness duration	0.14	0.17	−0.19	0.06
MCS-12	−0.20	< 0.05	**0.21**	**0.03**
PCS-12	0.13	0.20	−0.10	0.29
BDI-II	0.10	0.29	0.08	0.44
SCI overall burden	0.17	0.09	0.05	0.63
Stress symptoms	0.12	0.22	−0.01	0.89
Positive thinking	0.05	0.61	0.01	0.91
Active coping	−0.05	0.62	0.08	0.42
Social support	−0.13	0.18	0.08	0.39
Faith	−0.08	0.44	−0.04	0.72
Alcohol/smoking	0.04	0.69	−0.13	0.19

Age and education were used as covariates for all variables except age, for which only education was used. False discovery rate correction was used for multiple comparisons; the significant result is in bold. BDI-II, Beck Depression Inventory-II; MCS-12, Mental Component Summary of the SF-12; PCS-12, Physical Component Summary of the SF-12; SCI, Stress Coping Inventory.

## Discussion

The present study investigated 214 individuals with psoriasis (107 with illness duration ≤16 years and 107 with illness duration >16 years; cut-off: median) regarding their mental health in relation to illness duration. While individuals with illness duration ≤16 years had higher psoriasis severity, both groups had a low mean psoriasis severity, as indicated by low mean PASI scores, and low stress level with effective coping strategies, as shown by SCI subscales. The sample exhibited lower physical and mental health than the general population, as shown by lower SF-12 scores relative to the norm sample. Current psoriasis severity did not show any significant associations with mental health variables in either group. Age was positively correlated with physical health only among individuals with illness duration ≤16 years. The mean PASI score of the sample was low, likely because most participants were receiving treatment with biologics, and had a small degree of variance, discounting a few outliers – a condition not ideal for the performance of correlation analyses. Previous research has shown that patients treated with biologics are significantly more satisfied than those not treated with such therapy.^16^ Individuals with illness duration >16 years might have had a lower mean PASI score because of long-term continuous treatment at the outpatient centre or their lower smoking rates, as smoking is known to worsen psoriasis severity.^44^

The mean SF-12 scores of the current sample were below those listed in the SF-12 manual for the corresponding age group of individuals aged 45–54 years.^39^ While the publication date and geographical origin of the norm sample differ from those of the current sample, these results nonetheless suggest that individuals with psoriasis might experience decreased physical and mental health in comparison with healthy controls. This finding is in line with studies that have included patients with moderate-to-severe psoriasis.^15,16^ However, these results should be interpreted with caution, as this study was conducted during the COVID-19 pandemic, which may have contributed to a decline in mental health. Furthermore, instead of a contemporaneous control group, a prepandemic norm sample was used for comparison. Compared with prepandemic studies of individuals with psoriasis, including those with moderate-to-severe disease, the MCS-12 scores of individuals included in our study were lower.^45,46^ In addition, both groups had low mean BDI-II scores, with only a few individuals exceeding a score of 18, indicating clinically relevant depressive symptomatology. SCI scores suggested low general stress burden, few mental and physical stress symptoms and multiple effective coping factors, such as positive thinking, active coping and social support. All in all, apart from generally lower mental health, the current sample of individuals exhibited low psoriasis severity, few depressive symptoms low stress burden and effective coping strategies, independent of illness duration.

Interestingly, PASI did not correlate with mental health, stress variables or depressive symptoms, suggesting that mental state was not influenced by disease severity, and it did not exert an influence on severity. These findings do not reflect the results of other studies,^15,16,33,47^ although they are consistent with data from another recent study from our department. That study showed that the disease burden of psoriasis on relatives was independent of the patients’ disease severity and was instead linked to the patients’ susceptibility to psoriasis *per se*.^48^ Similarly, Sampogna *et al*.^49^ found weak correlations between PASI and SF-12 values in a sample investigating moderate-to-severe psoriasis, suggesting a revision of the PASI due to a limited validation and insufficient consideration of psychosocial impact. Therefore, rather than disease severity, factors not investigated in the current survey may have exerted stronger effects on mental health and may have contributed to the lower mental and physical health observed in the sample. This interpretation is further supported by the strict governmental measures in Austria during the COVID-19 pandemic, as several restrictions were in place at the time of the survey.^50^

More advanced age was associated with worse physical health in individuals with illness duration ≤16 years and >16 years, although this correlation did not remain significant after FDR correction in the latter group. This result was expected, as the number of somatic ailments typically increases with age, for example the higher prevalence of somatic comorbidities in the current sample. Additionally, age showed positive correlations with mental health in both groups, although these associations were not significant after FDR correction. Nonetheless, the moderate effect sizes highlight the importance of age as a possible protective factor in coping with psoriasis. This interpretation is supported by the differing correlation between MCS-12 and PASI: positive in individuals with illness duration >16 years and negative in individuals with illness duration ≤16 years.

Moreover, resilience is known to improve with age^51^ and may have enhanced influenced individuals’ ability to cope with the diagnosis, subsequently improving mental health. An investigation by Jankowiak and colleagues showed an association between lower self-consciousness and fewer feelings of unattractiveness and stigmatization.^52^ Interventions aimed at strengthening self-confidence may therefore be effective in increasing wellbeing and daily functioning, especially among younger individuals with psoriasis. Furthermore, the development of self-management, self-monitoring. and self-intervention skills has been highlighted as crucial for adapting to life with the disease.^53^

This study has several limitations. Firstly, there was no control group for direct comparison; therefore, the norm sample was used to compare SF-12 scores. Secondly, limited statistical power resulting from the modest sample sizes may have obscured meaningful relationships between the analysed parameters. Thirdly, the cross-sectional design precludes conclusions about causality. Fourthly, the mean PASI score was low and had little variance apart from a few outliers, leading to an impeded implementation of statistical analyses. As a result, correlation effect sizes may have been underestimated, and the statistical power of the Mancova may have been reduced. This probably reflects the high quality of specialized outpatient treatment at the study site. Finally, the results may not be generalizable, as the study was conducted during the COVID-19 pandemic in a well-treated patient sample undergoing treatment at a university hospital.

While current psoriasis severity did not significantly influence the mental health of individuals with psoriasis, or vice versa, the sample still exhibited reduced general mental health. On one hand, this may be a consequence of the COVID-19 pandemic and associated measures; on the other hand, individuals may be affected by the long-lasting psychological impact of psoriasis beyond singular illness episodes. Conclusively, individuals with both shorter (≤16 years) and longer (>16 years) illness duration require continued mental health support. Given the cross-sectional design and lack of a control group, further longitudinal and controlled investigations are warranted to clarify these associations.

## Data Availability

The data underlying this article will be shared on reasonable request to the corresponding author.
